# A Bibliometric Analysis of Scientific Publications on Cultural-Historical Psychology from 2010 to 2020: Dynamics, Geography, and Key Ideas

**DOI:** 10.11621/pir.2022.0412

**Published:** 2022-12-30

**Authors:** Boris G. Meshcheryakov, Victoria V. Ponomareva, Anna A. Shvedovskaya

**Affiliations:** a GBOU VPO of Moscow Region, Dubna State University, Dubna, Russia; b Moscow State University of Psychology and Education, Moscow, Russia

**Keywords:** Scientometric analysis, cultural-historical psychology, activity approach, L.S. Vygotsky, Web of Science, scientometrics

## Abstract

**Background:**

This paper presents the results of a study into the breadth, dynamics, and diversity of the interdisciplinary branch of cultural-historical psychology. The scatter of thematic areas within the cultural-historical approach indicates the urgent need to continue a systematic and holistic analysis of research related to cultural-historical topics in the context of its various directions and research groups.

**Design:**

A bibliometric analysis of scientific publications indexed by the Web of Science CC was carried out for the 2010–2020 period . Our previous bibliographic study (Rubtsov et al., 2019) revealed that the number of publications on cultural-historical psychology and citations of them, has recently increased, although unevenly.

**Results:**

According to our results, the number of publications on cultural-historical psychology is growing unevenly; publications from Russia and the United States made up almost equal shares of the sample, and third place was taken by England, followed by Finland and Sweden. The top 10 journals fell into two subject areas: Psychology and Education and Educational Research. With regard to the geographical location of the publishing houses of the top 10 journals, the highest number was taken by England and Russia. The dominant areas of research were teacher education, university education, and learning activity.

**Conclusion:**

The most frequently used terms were Vygotsky, activity approach, CHAT, CHP, ZPD, and learning activity.

## Introduction

The theoretical and methodological views of L.S. Vygotsky and his closest associates form a significant part of the foundation of scientific discourse and retain great heuristic power in modern psychology. In many ways, Vygotsky’s revolutionary thoughts modified the ideas about the unit of analysis at different stages of the formation of such directions in cultural-historical psychology as the theory of activity (Meshcheryakov & Ponomarev, 2018; Sannino & Engeström, 2018). But the perception of Vygotsky’s theory in the international academic communities varies a lot. In his critical analysis Dafermos (2016) noted that there are at least three widespread theoretical frameworks of interpretation of Vygotsky’s theory: cognitivism, culturalism, and cultural-historical activity theory. Many researchers emphasize significant differences in the traditions of developing ideas of cultural-historical psychology, in particular, between Russian and non-Russian authors (Bakhurst, 2016; Dafermos, 2016; Miller, 2011).

In modern works, three approaches (paradigms) are often described, compared, and partially juxtaposed (Ellis, Edwards, & Smagorinsky, 2010, Ch. 1, Introduction): 1) the cultural-historical, which has its roots primarily in Soviet-Russian psychology and philosophy; 2) the social-cultural, associated primarily with a number of famous North American and British psychologists, as well as Spanish authors (Valsiner, & Rosa, 2007, Editors’ Introduction); and 3) cultural-historical activity theory (CHAT), which is closely related to the school of activity theory in Helsinki, Finland (Sannino & Engeström, 2018).

Within this framework, it is interesting to look at the scientific mapping of the spread of Vygotsky’s ideas. This can be achieved through bibliometric research methods with focus on visualizing the structure and dynamics of the research field. We can use statistical methods to identify the outcomes of individuals or research groups, institutions and countries, and national and international networks, and map the development of the field of cultural-historical psychology. But there is a very small number of such bibliometric studies in the research field of cultural-historical psychology.

J. Valsiner pioneered measuring the annual frequency of citation of various works of Vygotsky in English-language publications and started with the 1969 to 1984 period. His focus was on analyzing differences in the citations of Vygotsky’s works, but he also recognized that “efforts to popularize Vygotsky’s name among English-speaking psychologists have succeeded to a great extent” (Valsiner, 1988, p. 156). W.M. Roth and Y.L. Lee (2007) demonstrated that the 1975–2005 period was marked by an impressive growth in citations and an increase in search results for the keyword “theory of activity.”

In terms of specific research fields, the bibliometric analysis has shown that Vygotsky’s ideas represent modern trends in educational research, specifically the use of dialogic teaching methods (Song et al., 2019), learning English as a second language (Zhang, 2020), creativity and education (Hernández-Torrano, & Ibrayeva, 2020), educational games (Liu et al., 2020), special education, disability, and inclusion (Bal et al., 2020). Another significant direction that has shown up in bibliometric analysis is the application of Vygotsky’s ideas to the study of human-computer interaction and digital technologies. T. Clemmensen, V. Kaptelinin, and B. Nardi found that there are different ways of using, adapting, and developing activity theory for different purposes. They refer to analyzing theory and developing new questions about it; defining requirements for new tools and supporting empirical analysis; and providing practical recommendations (Clemmensen et al., 2016). Using the bibliometric method, S. Karanasios, B. Nardi, C. Spinuzzi, and J. Malaurent ponted to the role of activity theory in the study of human-technology relations and such digital technologies as social media, smartphones, blockchain, artificial intelligence, and algorithmic decision-making (Karanasios et al., 2021).

In terms of applying the specific concepts of the cultural-historical approach, an increase in publications can be also seen; for example, between 2000 and 2019 the number of publications on the “zone of proximal development” (ZPD) grew (Margolis, 2020). A bibliometric mapping analysis of publications from Indonesia for the 2011-2020 period found that the keyword “activity theory” was among the six most-used keywords on the topic of Educational Technology (Darmawansah, 2021).

But it is important to understand that it is not always possible to trace the trajectory of the movement of scientific ideas, possible dead ends, or breakthrough directions from separate scientific publications. V. Zaretskii uses the image of a tree as Vygotsky sometimes did to illustrate his ideas. Publications are fruits, or finished products (Zaretskii & Nikolaevskaya, 2019). But can they always be used to reconstruct the process of obtaining them, or to identify the tree on which they grew, or the gardeners who tended them?

We need to look at the interconnections within the scientific schools. Authorial collaborations in scientific publications can give us an idea of the invisible colleges of scientific thought. An invisible college is a group of interacting scholars or scholars who share similar research interests in a subject area, who frequently produce publications related to that subject, and who communicate formally and informally with each other to achieve important goals on the subject, even though they may belong to geographically dispersed research affiliates (Zuccala, 2006).

### Background

The scatter of thematic areas within the cultural-historical approach indicates the urgent need to continue a systematic and holistic analysis of research related to cultural-historical topics in the broad context of its various directions and research groups.

Our previous bibliographic study (Rubtsov et al., 2019) revealed that the number of publications on cultural-historical psychology and their citations, has recently increased, although unevenly. The study’s total sample accounted for 5,669 works (published within 2009-2019 period) and included 1,817 publications from the Web of Science Core Collection and 2,838 from the RSCI database (Russian Science Citation Index). The sample consisted of publications containing the following Author Keywords: cultural-historical psychology (CHP sample) and Vygotsky (Vygotsky sample). The CHP sample embraced 181 publications, which included 161 scientific articles (88%). Most of these papers were in Russian (87% of the total). The total number of citations in these publications was 457; the h-index of the sample was 12. The Vygotsky sample appeared in 1,636 publications, of which 1,278 (78%) were scientific articles. Publications in Russian accounted for 10% of the total. The total number of citations for all works in the Vygotsky sample reached 7,850, and the h-index of the sample was 40. Let us look at this sample in greater detail.

The Vygotsky sample revealed that most publications included the keywords Activity (436 publications, or 32% of the total number in the sample), Tool (241 or 15%), and Zone of Proximal Development (226 or 14%). The analysis of the representation of publications in the Vygotsky sample by year showed that for Activity, the maximum number of publications was 72 in 2016; for Zone of Proximal Development, 32 and 33 (2015 and 2017, respectively); and for Tool, 40 publications (2017). The analysis of the geographical distribution of the groups of authors in this sample revealed that the United States had the largest number of publications, with 305 (18.64%), followed by Russia with 221 (13.51%), and Brazil with 162 (9.90%).

Our analysis of the terms used in the titles and abstracts of the publications in the Vygotsky sample identified four clusters of terms, which were classified by us: Cultural-Historical Psychology (1), Education (2), Development (3),and Zone of Proximal Development (4). Each had a different set of links between the terms. In cluster 1, the strongest links were between the following terms: psychology, L.S. Vygotsky, cultural-historical psychology, thinking, consciousness, speech, emotions, art, and personality; in cluster 2, learner/student, teacher, skills, tutor, case analysis, class, application, resource, and mathematics; in cluster 3, young child, parent, motive, early childhood education, self-regulation, family, speech, emotions, art, and literacy; in cluster 4, learner, scaffolding, developmental delay, language learning, and dynamic assessment.

In addition, the study presented the citation dynamics of various works and publications of L.S. Vygotsky for the 1999-2019 period based on a sample of 1,014 publications from Google Scholar. The dynamics of the number of citations of Vygotsky’s work showed an increase, with the peak in 2017, when it reached 24,226 citations per year. At the beginning of the analyzed period in 1999, a total of 2,724 citations were shown per year; in 2009 it was 12,396, and in 2018 it was 21,078. If we turn to the most cited works, we note that the five most-cited Vygotsky works included publications in English (two), Portuguese (one), and Russian (two). All the publications were books.

## Method

### Aim

This study was aimed at analyzing the thematic diversity of publication activity within the framework of the modern branch of cultural-historical psychology in the period from 2010 to 2020, taking into account certain bibliographic variables (year of publication, country, journal, university, and research field).

The following research questions were investigated through bibliometric mapping analysis:

RQ1: What were the dynamics of publications in cultural-historical psychology?

RQ2: Which countries, organizations, and journals have contributed to cultural-historical psychology-related research?

RQ3: What were the most-used keywords in the abstract sections of journals on cultural-historical psychology from 2010 to 2020?

RQ4: What was the semantic similarity of publications in different countries, universities, and journals on cultural-historical psychology from 2010 to 2020?

### Sample

The Web of Science Core Collection (hereinafter WOS CC) was the empirical base of the present study. Web of Science was selected as the scientific publication source in the study due to it having the largest research database.

### Data collection

The study sample consisted of scientific publications included in the WOS CC for the period of 2010-2020 (see *Table 1*).

**Table 1:** Sampling of publications in Web of Science Core Collection

N	Sample	Sampling
1	n = 105	Advanced search in the Web of Science Core Collection by the field AK=Author Keywords: Cultural-historical psychology with specification for compliance with the cultural-historical approach. Articles that directly indicate cultural-historical psychology formed the core (core sample records) of the publication for this study. The query formulation in the Web of Science Core Collection advanced search: AK=((“Cultur* Histor* Psychol*”) or (“Cultur* Histor* Activ* Theor*”)) and PY=(2010-2019).
2	n = 526	Analysis of the Web of Science Core Collection citation report, followed by a transition to an array of publications citing core sample records (n=105). Selection of publications that do not belong to the core sample.
3	n = 446	An expert assessment of the sample of publications citing the core with confirmation of the relevance of this sample to the subject of cultural-historical psychology and the activity approach.
4	n = 551	Combination of the core samples with the publications citing the core. Thus, a combined sample of core+ was obtained. The conducted analysis covered the publications for the 11-year period (2010-2020).

### Data analysis

The methodology of the present study primarily involved a bibliographic analysis across scientific publications to test the study hypothesis about the thematic heterogeneity of cultural-historical psychology and the activity approach.

Bibliometric analysis of publications involved the tools of the Web of Science platform. Bibliometric analysis of publications of the core and the core+ was carried out according to the following parameters: year of publication, country, source of publication, scientific organization, and research field.

The thematic diversity of the content of the core+ sample was analyzed on the basis of the Author Keywords. The Author Keywords are indicated in the articles in the relevant section of the publication. For the analysis, the Author Keywords were used in their original version (without changes). A free software VOSviewer v.1.6.13 was used to process the information, received in the Web of Science through co-word analysis of the text, and to visualize the relationships among the Author Keywords. To combine expressions that were close in meaning, a VosViewer Thesaurus File was used, which takes into account the synonymy of the Author Keywords and unifies the representation of the plural and singular (analysis of co-occurence). When creating a visual map of terms, a threshold for frequency of occurrence was set at 5, which corresponds to 61 keywords (*Figure 2)*.

For quantitative analysis of the frequency profiles (distributions) of terms, the following mathematical statistical methods were used (SPSS Statistics 23.0): calculation of matrices of linear correlation coefficients r (Pearson product-moment method); and then, for comparison, calculation of distances by formula: d = 10 (1 – r). If d = 0, we can say that the frequency profiles of the terms were completely similar (with r = +1); the possible maximum value is 20 (if r = –1), but in the overwhelming majority of cases, d did not exceed 10 units. Terms are semantic units. The distances were calculated between the frequency distributions of these terms (semantic units). These distances will be referred to as semantic distances.

The use of hierarchical cluster analysis (distances were also estimated on the basis of correlation) made it possible to quantitatively and visually assess the degree of semantic similarity or the distance of the compared samples.

In order to clarify the conclusions based on cluster analysis, factor analysis (principal component analysis) was also carried out by orthogonal Varimax rotation using Kaiser normalization. Here, it should be noted that the described method of analysis did not constitute a variant of co-word analysis (Callon, Courtial, & Laville, 1991), since it was not based on estimates of the strength of links in pairs of keywords co-occurring in publications, and its units of analysis were frequency profiles of terms in specially organized subsamples of publications.

## Results and Discussion

The thematic diversity analysis of cultural-historical psychology and activity approach was carried out in two directions: analysis of the frequency distributions of publications in two samples (core and core+) in relation to the year of publication, countries, and journals; and analysis of the thematic diversity of publications related to the core+ sample in relation to the countries, journals, organizations, and scientific fields.

### Frequency distribution analysis

RQ1: What were the dynamics of publications in cultural-historical psychology?

In order to explore the development of interest in cultural-historical issues in terms of its sporadic or uniform character, we examined how many articles related to the topic were published annually in editions indexed by the Web of Science Core Collection from 2010 onwards. The publication dynamics of the core and core+ samples were evaluated separately.

**Figure 1.**
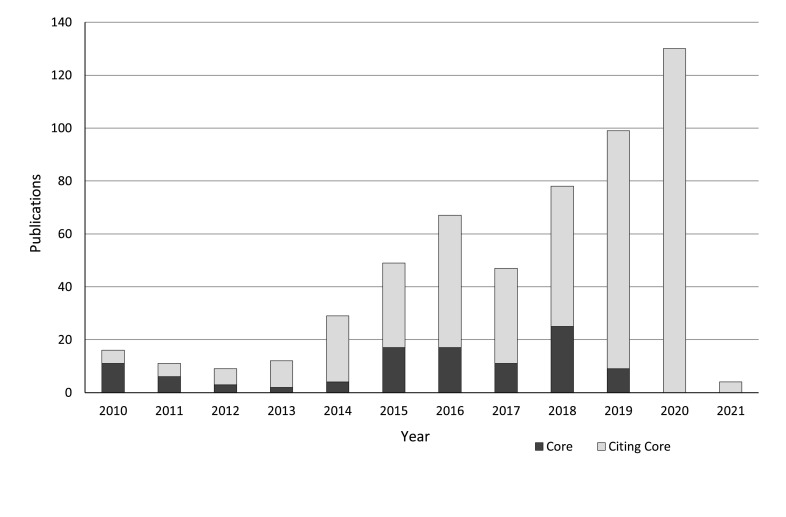
Distribution by year of publications in the core+ sample (n = 551, according to the Web of Science Core Collection): publications related to the core sample (n =105, according to the Web of Science Core Collection), publications citing core sample (n = 446, according to the Web of Science Core Collection).

The dynamics of the publication activity of the core+ are presented below (*Figure 1*).

The sporadic nature of publication activity can be identified in terms of frequency distribution of core publications by year, with the largest number of publications (24%) appearing in 2018. More broadly, the lowest publication activity is observed from 2011 to 2014 (14%), while the period 2015–2019 accounts for the majority of publications (75%).

The nature of the distribution of publications in the core+ sample, including publications of the core and core+ sets, corresponds to the overall pattern of publication and citation in the social sciences: in general, articles started to be actively cited around three to four years after their publication. In this connection, there was an annual increase in the number of publications constituting the core+ sample: the highest number of publication citations appeared in 2020 (n = 130), while the lowest number was in 2012 (n = 9). The data for 2021 are not included in this analysis since 2021 has not yet ended, and some works are yet to be published.

Therefore, it can be seen that the number of publications on the cultural-historical psychology is growing unevenly. The annual number of core publications increased by 2.5 times, and the number of core+ by more than eight times.

RQ2: Which countries and journals have contributed to cultural-historical psychology related research?

Analysis of the affiliation of the authors by sample showed the geographical spread of the cultural-historical approach in terms of the 10 top countries in which the research was carried out. *Table 2* demonstrates the frequency distribution of publications in the core+ sample by the authors’ country affiliations.

**Table 2:** Top 10 countries by number of publications included in the core+ sample (n=551, according to Web of Science Core Collection)

	Countries	Number of publications	Number of publications (% of 551)
1	Russia	96	17.42
2	USA	95	17.24
3	England	52	9.44
4	Spain	33	5.99
5	Australia	27	4.90
6	Canada	26	4.72
7	Brazil	24	4.36
8	Finland	22	3.99
9	Norway	22	3.99
10	Sweden	21	3.81

Almost equal shares of the core+ sample are taken by publications from Russia and the United States (17.42% and 17.24%, respectively). The third place is taken by the group of authors affiliated with England (9.44%). Finland and Sweden entered the top 10 in terms of the number of publications in the core+, while Germany and Bulgaria failed to meet the threshold (see *Table 2*).

It is interesting to look at the specific academic journals which published works on cultural-historical topic (*see Table 3*).

**Table 3:** Top 10 journals by number of publications included in the core+ sample (n=551, according to Web of Science Core Collection)

	Journal Title ISSN, Publisher, Country, research Theld in WoS CC	Number of publications	Number of publications (% of 551)
1	Cultural-Historical Psychology ISSN: 1816-5435, MSUPE, Russia (Psychology)	40	7.26
2	Educational Studies in Mathematics ISSN: 0013-1954, Springer, Netherlands (Education & Educational Research)	16	2.90
3	Issues of Psychology (Voprosy psikhologii) ISSN: 0042- 8841, International Book, Russia (Psychology)	16	2.90
4	Learning Culture and Social Interaction ISSN: 2210-6561, Elsevier, England (Education & Educational Research)	15	2.72
5	Mind, Culture and Activity ISSN: 1074-9039, Taylor & Francis, England (Education & Educational Research)	12	2.18
6	Psychological Science and Education ISSN: 1814-2052, MSUPE, Russia (Psychology)	10	1.82
7	Theory & Psychology ISSN: 0959-3543, Sage, England (Psy- chology)	10	1.82
8	ZDM Mathematics Education ISSN: 1863-9690, Springer, Germany (Education & Educational Research)	10	1.82
9	Frontiers in Psychology ISSN: 1664-1078, Frontiers Media, Switzerland (Psychology)	7	1.27
10	Infancia y Aprendizaje ISSN: 0210-3702, Taylor & Francis, England (Psychology)	7	1.27

Analysis of the periodicals publishing articles of the core+ sample showed that 10 journals were responsible for more than 25% of publications. Among these, the leader in terms of the number of publications was the *Cultural-Historical Psychology* journal (7.26%); second place was shared by the journals *Educational Studies in Mathematics* and *Issues of Psychology* (2.9% each). Third place in terms of the share of publications was taken by the *Learning Culture and Social Interaction* journal (2.72%). The top 10 journals pertained to two subject areas: Psychology (six journals) and Education & Educational Research ( four journals). These journals are published by Springer (Netherlands), Taylor & Francis (England), and MSUPE (Russia) (2 journals each). With regard to the geographical location of publishing houses of the top 10 journals, the largest numbers were in England and Russia (four and three journals, respectively).

Thus, in the first part of the analysis of our research results, we distinguished the groups of countries and academic journals having the largest number of publications in cultural-historical psychology, and determined the yearly frequency of these publications.

RQ3: What were the most-used keywords in the abstract sections in journals on cultural-historical psychology from 2010 to 2020?

The frequencies of the representation of Author Keywords in the sample were calculated in order to analyze the content of publications related to cultural-historical psychology. A semantic analysis of the 1,742 words and phrases (author keywords) contained in the sample was carried out. Keywords that were close in meaning were combined; these were those which might differ in singular or plural, presence or absence of articles, spelling errors, or they may contain synonyms. Among the Author Keywords there were unspecific terms for cultural-historical psychology. By referring in the same publication to terms related to the field of cultural-historical psychology (CHP) or to the cultural-historical activity theory (CHAT), they showed the intensity of work in a particular field of cultural-historical research. Semantic analysis resulted in a sample of 1,532 keywords.

*Table 4* shows that of the 20 most frequent terms, the first three places are occupied by: CHP — 86 articles; activity approach — 55; and CHAT — 50. The keywords indicated the following research fields to be prevalent: teacher education, university education, and learning activity. Development, subjectivity, reflection, and identity were among them, as well as specific terms in cultural-historical psychology — ZPD, perezhivanie, and double stimulation.

**Table 4:** Top 20 terms (keywords) by frequency of occurrence (according to the Web of Science Core Collection)

	Term (Examples of variants it encodes)	Frequency of occurrence of the term
1	CHP (cultural-historical psychology, Vygotsky’s theory, cultural-his- torical scientific school, historical-cultural psychology, cultural-historical approach)	86
2	activity approach (activity principle, activity theory, theory of activity)	55
3	CHAT (cultural-historical activity theory)	50
4	Vygotsky (Vygotski, Lev Semenovich Vygotsky, etc.)	38
5	teacher education	24
6	ASD (autism spectrum disorders, autism spectrum condition, autism, etc.)	19
7	ZPD (zone of proximal development, proximal development zone, etc.)	18
8	learning activity	16
9	education	14
10	development	13
11	university education	12
12	perezhivanie (feeling, experiencing)	12
13	subjectivity (subjetividad)	11
14	contradictions	11
15	methodology	10
16	reflection (reflexivity)	10
17	double stimulation	10
18	identity	10
19	sociocultural theory (sociocultural approach, sociocultural perspec- tive)	9
20	social interaction	9

**Figure 2.**
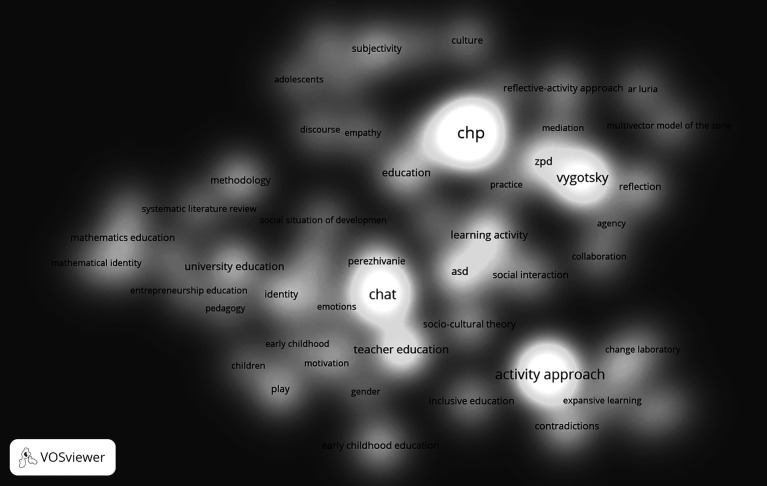
Map of relationships between Author Keywords in publications of the core+ sample (n = 551, according to the Web of Science Core Collection)

The representation of the core+ keywords can be visualized using VosViewer tools. The size of a term on the map of keywords below (*Fig. 2*) is determined by the frequency of the keyword use. The density of keywords placement depends on the number and intensity of links between them. On this keyword map, the lighter the area around a keyword, the higher the frequency of its use. The density of placement of keywords depends on the number and strength of links between them.

As shown in *Figure 2*, such Author Keywords as CHP (o = 86; ls = 138), Activity approach (o = 55; ls = 63), CHAT (o = 50; ls = 51), and Vygotsky (o = 38; ls = 69) were the most represented for the core + sample in terms of the number of uses (weight<occurrences>, o hereafter) and the total weight of the links (weight<total_ link_strength>, ls hereafter). Also the top 10 of the most used keywords included Teacher Education (o = 24; ls = 33); ZPD (o = 18; ls = 43); Autism Spectrum Disorders (ASD) (o = 19; ls = 15); Learning Activity (o = 16; ls = 24); Development (o = 13; ls = 39); and Education (o = 14; ls = 32).

Unfortunately, not all publications included in the analysis of thematic diversity contained complete bibliographic data (according to the Web of Science Core Collection). For example, Author Keywords might have been missed in some publications. For this reason, our analysis omitted publications in one of the most significant scientific journals, *Mind, Culture, and Activity*.

Since many of the 1,532 samples were not specific to a particular scientific field, and 78% had a low frequency of occurrence (=1), the next step was to select an abbreviated list of terms for subsequent analysis. The selected Author Keywords were contained in a sample of those articles where “Cultural-historical psychology” and “Cultural-historical activity theory” (that is, core publications) were used among the full list of the Author Keywords. Thus, the sample consisted of 368 keywords (Shvedovskaya, 2021).

RQ4: What was the semantic similarity of publications on cultural-historical psychology in different countries, universities, journals from 2010 to 2020?

This study analyzes the semantic distances between different samples of publications, differentiated by a number of variables: country, university, journal, and research field. Semantic proximity is understood as the distance between frequency distributions of terms in the publications.

Subsequent analysis involved the top 20 most frequently used keywords (*see Table 4*) to identify the relationship (semantic distances) between the most productive countries (n = 10), organizations (n = 10), sources (n = 10), and subject areas (n = 10) in publications where they were used.

**Figure 3.**
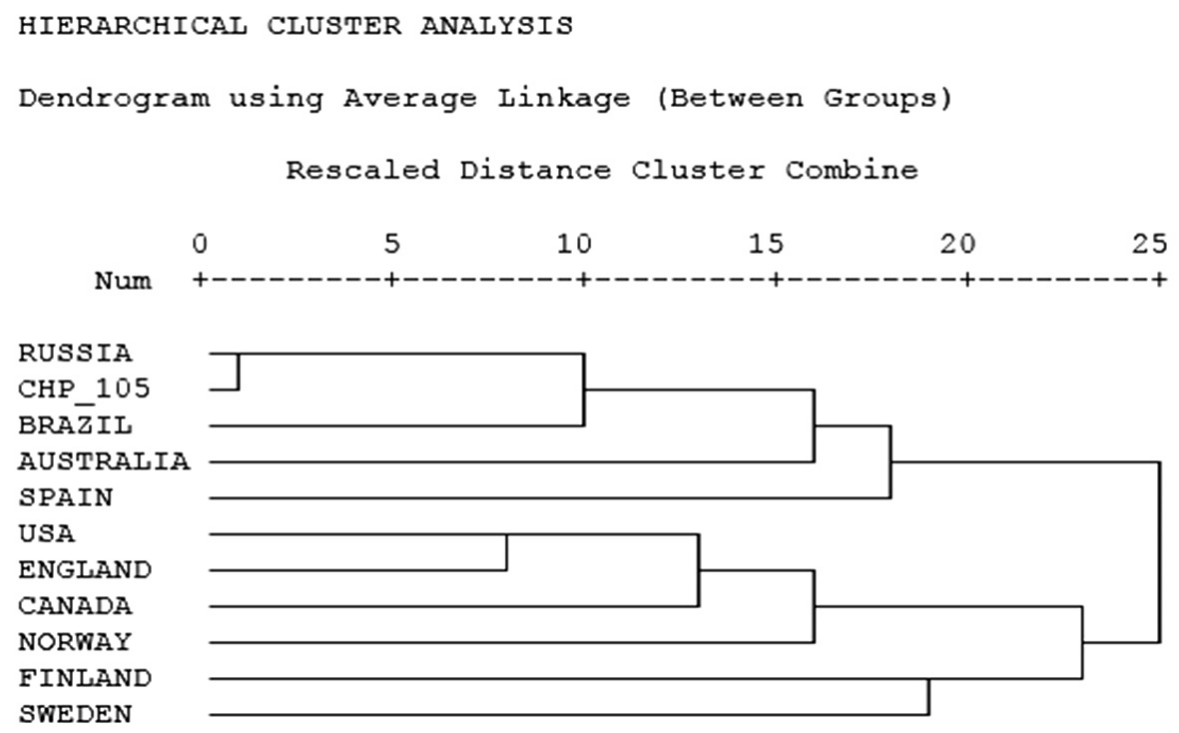
Dendrogram for samples of publications in different countries

*Table 5* and *Figure 3* present a matrix of the distances between the samples of publications, grouped by country. In total, the analysis included samples from 10 countries in Europe, Australia, and North and South America. The average semantic distance between all countries was 6.98.

**Table 5:** Semantic distances between the samples of publications according to the geographic affiliation of the authors, and their distances from the core (CHP_105) and core+ (CHP_551) samples

Country	Brazil	Australia	USA	Spain	Canada	England	Norway	Sweden	Finland	CHP_551	CHP_105
Russia	3.86	4.85	7.02	5.66	8.17	8.33	9.72	7.86	9.01	1.97	1.41
Brazil		6.56	8.28	5.74	8.73	9.40	9.16	8.67	9.73	4.11	3.52
Australia			5.02	6.49	5.08	6.54	8.06	8.01	8.01	3.43	4.25
USA				6.04	3.95	3.24	4.85	6.87	6.62	2.86	4.55
Spain					7.46	8.00	7.94	8.25	7.93	4.00	4.99
Canada						5.25	5.70	7.78	8.07	4.77	5.70
England							5.15	5.08	6.80	4.21	6.53
Norway								6.60	8.54	6.16	6.79
Sweden									5.92	5.43	7.12
Finland										6.40	8.56

As can be seen from *Figure 3*, the analysis of semantic distances of terms by geographical affiliation of the authors showed that the closest to each other were the United States and England (d = 3.24); Russia and Brazil (d = 3.86); and the United States and Canada (d = 3.95) (see *Table 5*). On the other hand, the greatest distances were between Brazil and Finland (d = 9.73); Russia and Norway (d = 9.72); and Brazil and England (d = 9.40).

The dendrogram (*Figure 3*) obtained for countries with a cut close to the root clearly shows two large clusters. The first of these clusters includes Russia, Brazil, Australia, and Spain (average d between them = 5.53). The second cluster includes the countries of North America, as well as England and the Scandinavian countries (average d in the second cluster = 6.03). It should be noted that the geographical proximity of countries does not correlate with their thematic proximity. For example, neighboring Scandinavian countries differed thematically from each other much more markedly than the pair Russia-Brazil or the pair United States-England (*see Table 5*).

On the other hand, the same dendrogram, cut at a height of 20, depicts a differentiation of three clusters: the first remains the same, while the second includes the United States, England, Canada, and Norway (average distance = 4.69), and the third includes publications by Finnish and Swedish authors (distance = 5.92).

In order to confirm the correct selection of clusters, a factor analysis was carried out with a factor loading refinement of less than |0.25|. The analysis confirmed the presence of three components with eigenvalues (Ev) greater than 1: Ev1 = 4.46; Ev2 = 1.92; Ev3 = 1.06. Factor 1 included countries with the following factor loadings: Russia — 0.905; Brazil — 0.824; Spain — 0.610; Australia — 0.578; United States — 0.287. Factor 2: Australia — 0.466; Canada — 0.805; United States — 0.795; Norway — 0.728; England — 0.713. The core had factor loadings of 0.864 for factor 1 and 0.352 for factor 2. Factor 3: England — 0.430; Finland — 0.818; Sweden — 0.782. If a country is included in several components, it is assigned to a greater factor loading. Rotation transformed in four iterations.

We note that three terms were common to all groups of countries: activity approach, teacher education, and CHAT (see *Table 6*). Five more terms common to paired country groupings were CHP, Vygotsky, ZPD, contradictions, and university education. The first group of countries was responsible for 50.0% of repeated terms, the second group 66.7%, and the third 41.7%. Of course, this indicates a significant conceptual overlap with publications in different groups of countries. Without even knowing which countries are included in each group, the semantic analysis of the terms indicates that the first and third groups are associated with the Russian and Finnish schools of thought, respectively. These are united by three general terms, which are common to all three groups. Thus, it turns out that the middle group has the greatest number of overlaps with both the Russian school (six) and the Finnish school (five), which could be interpreted as its mixed character.

**Table 6:** Most common terms in publications by country group

Russia, Brazil, Spain, Australia	Canada, USA, Norway, England	Finland, Sweden
CHP	CHAT	activity approach
activity approach	activity approach	double stimulation
Vygotsky	Vygotsky	intervention research
subjectivity	teacher education	computer-based training
ZPD	ASD	formative interventions
education	ZPD	CHAT
perezhivanie	identity	teacher education
development	sociocultural theory	contradictions
reflection	contradictions	University education
reflective-activity approach	CHP	methodology
teacher education	play	collaboration
CHAT	University education	Clinic of Activity

**Figure 4.**
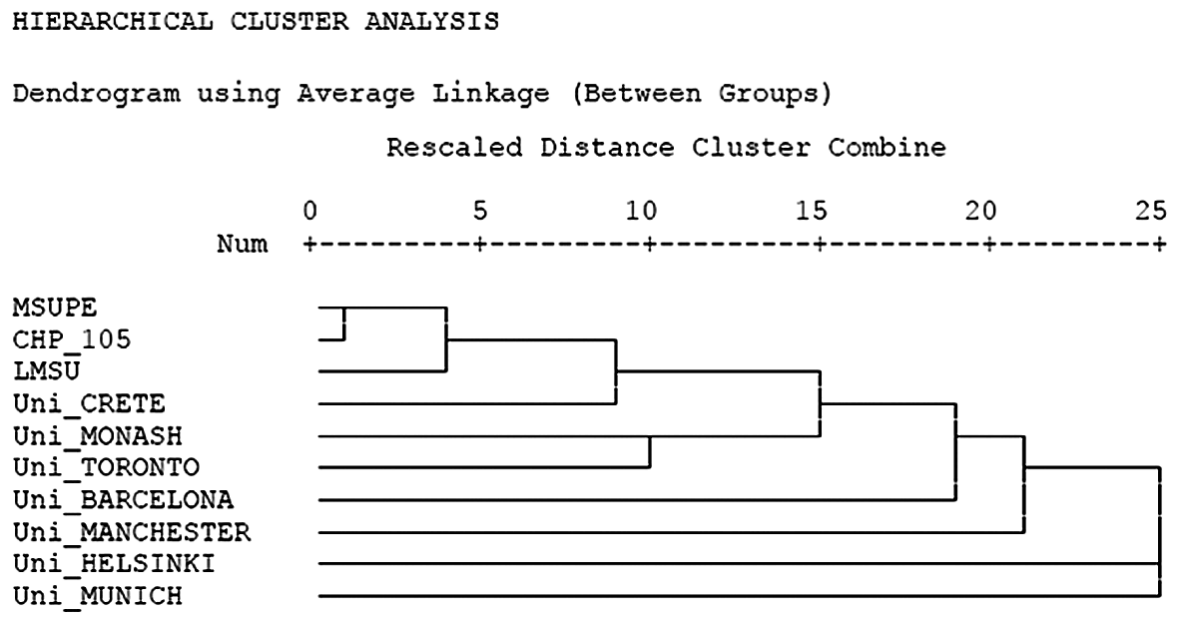
Dendrogram for samples of publications by the authors’ affiliation to different universities

Next, let us consider the distance matrices (see *Table 7*) and dendrogram (*Figure 4*) for the samples of publications differentiated by the authors’ affiliation to universities. The average distance between all universities was 8.09.

**Table 7:** Semantic distances between samples according to the authors’ affiliation to different universities, and their distances from the complete and core samples

Universities	Lomonosov Moscow State University	University of Crete	Monash University	University of Barcelona	University of Manchester	University of Toronto	University of Helsinki	University of Münich	CHP_551	CHP_105
Moscow State University of Psychology and Education (MSUPE)	3.36	5.01	5.41	7.04	9.23	8.22	9.33	9.44	2.47	2.12
Lomonosov State University Moscow (LMSU)		5.20	6.02	7.11	9.67	8.50	10.00	10.27	3.90	2.74
University (Uni_CRETE) of Crete			5.26	7.91	7.17	6.45	8.40	8.62	3.45	3.63
Monash (Uni_MONASH) University				8.48	6.55	4.84	8.87	9.48	4.25	4.56
University of Barcelona (Uni_BARCEL)					9.55	9.71	10.09	10.08	5.82	6.09
University of Manchester (Uni_MANCH)						8.42	8.82	9.25	6.04	7.31
University of Toronto (TORONTO)							10.13	9.60	7.35	7.52
University of Helsinki (Uni_HELSINKI)								9.89	7.05	8.73
University of Münich (Uni_MÜNICH)									8.07	9.00

As can be seen from *Figure 4*, the analysis of the semantic distances of terms according to the authors’ affiliation to different universities showed that the following universities are the closest to each other: Moscow State University of Psychology and Education and Lomonosov Moscow State University (d = 3.36); Monash University and the University of Toronto (d = 4.84); Moscow State University of Psychology and Education and the University of Crete (d = 5.01) (see *Table 7*). On the other hand, the largest distances were between Lomonosov Moscow State University and the University of Münich (d = 10.27); the University of Toronto and the University of Helsinki (d = 10.13); and the University of Barcelona and the University of Helsinki (d = 10.09).

The dendrogram shows that publications by authors affiliated with the University of Helsinki and Ludwig Maximilian University of Munich hold a special place among the publications of all university affiliations. The other seven universities form one large cluster (together with the core): Moscow State University of Psychology and Education (MSUPE), Lomonosov Moscow State University (LMSU), the University of Crete, Monash University, the University of Barcelona, the University of Toronto, and the University of Manchester (average d within the cluster = 7.10). Although the average distance between the publications of the University of Helsinki and all other universities was 9.44 (with the University of Munich — 9.58), they are also separated from each other by a significant semantic distance of 9.89.

These results also provide a basis for identifying at least three thematic groups within the analyzed sample of publications. This is consistent with the results of factor analysis with a factor loading refinement of less than |0.25|. The analysis confirmed the presence of three components with eigenvalues (Ev) greater than 1: Ev1 = 3.70; Ev2 = 1.35; Ev3 = 1.05. Factor 1 included organizations with the following factor loadings: Moscow State University of Psychology and Education — 0.845; Lomonosov Moscow State University — 0.842; the University of Barcelona — 0.582; the University of Crete — 0.563; Monash University — 0.386. Factor 2: the University of Crete — 0.459; the University of Toronto — 0.853; Monash University — 0.728; the University of Manchester — 0.464. The core had factor loadings of 0.863 for factor 1 and 0.301 for factor 2. Factor 3: the University of Crete — 0.304; the University of Helsinki — 0.802; the University of Manchester — 0.518; the University of Münich — 0.390. If an organization was included in several components, it was assigned to a greater factor loading. Rotation converged in four iterations.

If the university samples of publications are grouped according to the factors having the highest loadings, then the first group will include two Moscow universities (MSUPE, LMSU) and the universities of Barcelona and Crete, and the second group will comprise the University of Toronto and Monash University, while the third group will be made up of the universities of Helsinki, Manchester and Munich.

The thematic diversity and at the same time the overlap of factorized groups of publications, are illustrated in the table of the most frequently used terms in each group (see *Table 8*). All groups of universities have one common term (Vygotsky), while six terms are common for two groups of universities (CHP, development, ZPD, identity, dialectics, and contradictions).

**Table 8:** Most common terms in publications of universities

MSUPE, LMSU, Universities of Barcelona and Crete	University of Toronto, Monash University	Universities of Munich, Manchester and Helsinki
CHP	Vygotsky	double stimulation
Vygotsky	CHP	CHAT
activity approach	perezhivanie	activity approach
development	ZPD	ASD
reflective-activity approach	AR Luria	social interaction
reflection	early childhood	identity
education	emotions	Vygotsky
ZPD	development	intervention research
multivector model of ZPD	CHAT	dialectics
teacher education	dialectics	contradictions
social situation of development	double stimulation	leading activity
mediation	collaboration	formative interventions

*Table 8* shows that groups of organizations are semantically similar to groups of countries (see *Table 6*). This is because the parameters “organization” and “country” are related, as they are attributes of the authors of publications in the same sample.

We note that the term Vygotsky was used by all three groups of universities. CHP, Development, and ZPD were included in two groups: “MSUPE, LMSU, Universities of Barcelona and Crete” and “University of Toronto, Monash University.” Activity approach was used by “MSUPE, LMSU, Universities of Barcelona and Crete” and “Universities of Munich, Manchester and Helsinki.” CHAT was used by “University of Toronto, Monash University” and “Universities of Munich, Manchester, and Helsinki.” The remaining terms fell into one group only (see *Table 8*).

**Figure 5.**
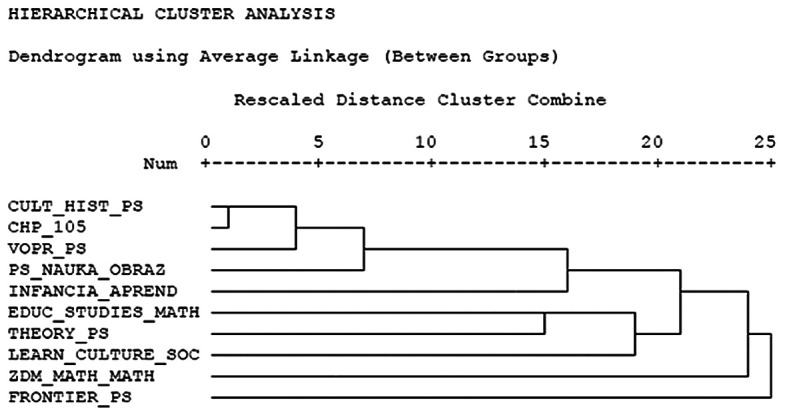
Dendrogram for samples of publications in different journals

Let us analyze the data on the samples of publications corresponding to different journals (*see Table 9* and *Figure 5*). The average distance between all pairs of samples amounted to 8.34.

**Table 9:** Semantic distances between the samples of publications corresponding to the top 10 journals

Journals	Issues of Psychology	Psychological Science and Education	Theory & Psychology	Infancia y Aprendizaje	Educational Studies in Mathematics	ZDM Mathematics Education (Zdm)	Frontiers in Psychology	Learning Culture and Social Interaction	CHP_551	CHP_105
Cultural-Historical Psychology (CULT_HIST_PS)	2.84	3.72	7.44	6.99	8.72	10.32	9.86	8.09	2.26	1.71
Issues (VOPR_of Psychology PS)		5.05	8.52	7.15	9.59	9.8	9.85	9.4	3.97	2.58
Psychological Science and Education (PS_NAUKA_OBRAZ)			6.82	6.78	7.76	9.58	8.92	8.98	2.42	2.99
Th(THEORY_eory & Psychology PS)				9.21	6.35	8.86	9.7	6.78	5.33	6.17
Infancia y Aprendizaje (INFACIA_APREND)					8.63	10.25	9.96	10.05	6.32	6.39
Educational Studies (EDUC_in Mathematics STUDIES_MATH)						6.53	9.76	8.35	4.99	6.72
ZDM Mathematics Education (Zdm MATH_MATH)							10.32	9.64	7.72	7.91
Frontiers (FRONTIER_in Psychology PS)								9.7	9.96	8.23
Learning Culture and Social Interaction (LEARN_CULTURE_SOC)									7.02	8.39

As can be seen from *Table 9*, the analysis of semantic distances by journals with publications included in the core+ sample showed that the following journals were the closest to each other: *Cultural-Historical Psychology* and *Issues of Psychology* (d = 2.84); *Cultural-Historical Psychology* and *Psychological Science and Education* (d = 3.72); *Issues of Psychology* and *Psychological Science and Education* (d = 5.05). On the other hand, the largest distances were between *Cultural-Historical Psychology* and *ZDM Mathematics Education* (d = 10.32); *ZDM Mathematics Education* and *Frontiers in Psychology* (d = 10.32); *Infancia y Aprendizaje* and *ZDM Mathematics Education* (d = 10.25).

The dendrogram in *Fig. 5*, cut at a height of 20 or so, is divided into two non-single clusters (four and three journals each) and two single journals. The first cluster with an average distance (d = 5.42) included three Russian journals (*Cultural-Historical Psychology, Issues of Psychology, Psychological Science and Education*), and a Spanish journal (*Infancia y Aprendizaje*). Another cluster (average d = 7.16) included *Educational Studies in Mathematics* (EDUC_STUDIES_MATH), *Learning Culture and Social Interaction* (LEARN_CULTURE_SOC_INT), and *Theory & Psychology* (THEORY_PS). Although the journals *ZDM Mathematics Education* (ZDM_MATH_EDUC) and *Frontiers in Psychology* (FRONTIERS_PS) have a specific thematic content, they were very semantically distant from each other (d = 10.32).

The results of clustering were consistent with the results of factor analysis with a factor loading refinement of less than |0.25|. The analysis confirmed the presence of three components with eigenvalues (Ev) greater than 1: Ev1 = 3.54; Ev2 = 1.52; Ev3 = 1.09. Factor 1 included journals with the following factor loadings: *Cultural-Historical Psychology* — 0.882; *Issues of Psychology* — 0.848; *Psychological Science and Education* — 0.766; *Infancia y Aprendizaje* — 0.528; Factor 2: *ZDM Mathematics Education* — 0.812; *Educational Studies in Mathematics* — 0.770; *Theory & Psychology* — 0.389. The core has factor loadings of 0.897 for factor 1 and 0.259 for factor 2. Factor 3: *Learning Culture and Social Interaction* — 0.786; *Theory & Psychology* — 0.634; *Frontiers in Psychology* — 0.376. If a journal was included in several components, it was assigned to a greater factor loading. Rotation transformed in four iterations.

*Table 10* shows the most common terms corresponding to three groups of journals. Two terms were included in all three groups of journals (activity approach, Vygotsky), while five more terms were common for pairs of groups of journals (ZPD, Development, learning activity, Teacher education, and CHAT).

**Table 10:** Most common terms in publications of three groups of journals

Psychology, Cultural-Historical Issues of Psychology, Psychological Science and Education, Infancia y Aprendizaje	ZDM Mathematics Education, Educational Studies in Mathematics	Learning Culture and Social Interaction, Theory & Psychology, Frontiers in Psychology
CHP	CHAT	double stimulation
activity approach	teacher education	Vygotsky
Vygotsky	review of the literature	CHAT
ZPD	identity	activity approach
Development	activity approach	ASD
education	Vygotsky	practice
reflection	methodology	play
reflective-activity approach	activity	dialectics
learning activity	leading activity	learning activity
teacher education	development	formative interventions
joint activity	discourse	intervention research
AR Luria	contradictions	ZPD

Analysis of the table showing the most frequent terms in these three groups of journals reveals a somewhat similar picture to that of the previous two analyses (for countries and universities): the first group has four common terms with the second group and three with the third; the second and third groups have three common terms. Therefore, we can again highlight the mixed character of the middle group and the difference in scientific schools in the first and third groups.

**Figure 6.**
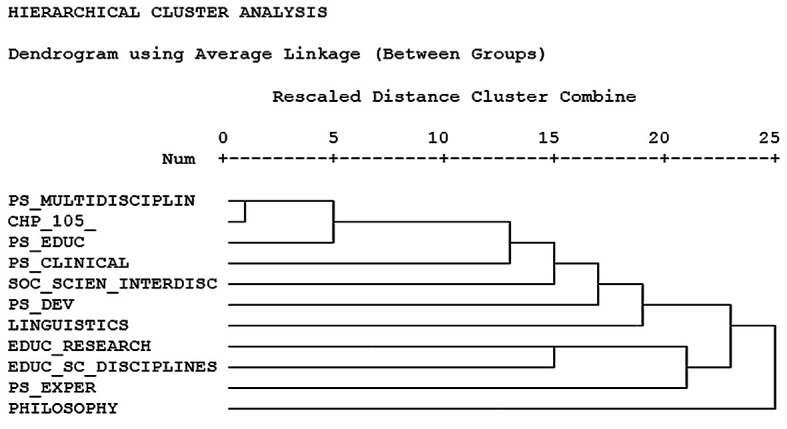
Dendrogram for samples of publications pertaining to different subject areas

Finally, let us analyze the data on distances and clusters within the samples of publications, distributed by so-called “subject areas” (*see Table 11* and *Fig. 6*). Here, the average distance between all pairs of samples was 7.87.

**Table 11:** Semantic distances between samples differentiated by subject areas and their distances from the total and core samples

Subject areas	Psychology, Educational	Psychology, Clinical	Education & Educational Research	Social Sciences, Interdisciplinary	Linguistics	Psychology, Developmental	Education, Scientific Disciplines	Psychology, Experimental	Philosophy	CHP_551	CHP_105
Psychology, Multidisciplinary (PS_MULTIDISCIPLINARY)	2.86	4.72	6.51	4.70	5.55	6.57	9.43	8.29	8.08	1.22	1.21
Psychology, Educational (PS_EDUC)		5.82	7.75	6.06	6.94	4.43	9.94	9.83	9.02	3.22	2.53
Psychology, Clinical (PS_CLINICAL)			9.53	7.46	8.49	8.19	9.63	10.40	9.17	5.70	4.92
Education & Educational Research (EDUC_RESEARCH)				7.25	6.30	7.07	5.92	6.76	8.73	2.79	4.94
Social Sciences, Interdisciplinary (SOC_SCIEN_INTERDISC)					7.10	6.88	9.91	7.46	8.48	4.45	5.07
Linguistics (LIN- GUISTICS)						8.99	9.02	8.21	10.15	4.76	5.59
Psychology, Developmental (PS_DEV)							9.95	8.94	10.15	5.69	6.24
Education, Scien-tific Disciplines (EDUC_SC_DISCIPLINES)								8.53	8.73	7.48	8.00
Psychology, Experimental (PS_ EXPER)									10.13	6.83	8.08
Philosophy (PHILOSOPHY)										8.04	8.69

As can be seen from *Figure 6*, the analysis of semantic term distances by the thematic directions to which publications in the Web of Science Core Collection were assigned, showed that the following areas were the closest to each other: Psychology, Multidisciplinary and Psychology, Educational (d = 2.86); Psychology, Educational and Psychology, Developmental (d = 4.43); Psychology, Multidisciplinary and Social Sciences, Interdisciplinary (d = 4.70) (*see Table 11*). On the other hand, the greatest distances were between Psychology, Developmental and Philosophy (d = 10.15); Linguistics and Philosophy (d = 10.15); Psychology, Clinical and Psychology, Experimental (d = 10.40).

If we cut the dendrogram (*Fig. 6*) slightly above level 20, we obtain a structure consisting of three clusters. The first cluster includes six subject areas (average d = 6.32) and a core sample; the second cluster includes three areas (average d = 7.07). Thethird cluster includes the thematic area of Philosophy almost equidistantly from the other two clusters (average d from the first cluster = 9.18; from the second, d = 9.20).

The results of factor analysis with a factor loading refinement less than |0.25| provide a basis for identifying a three-factor structure. The analysis confirmed the presence of three components with eigenvalues (Ev) greater than 1: Ev1 = 4.05; Ev2 = 1.52; Ev3 = 1.11. Factor 1 included subject areas with the following factor loadings: Psychology, Multidisciplinary — 0.884; Psychology, Educational — 0.869; Psychology, Clinical — 0.661; Social Sciences Interdisciplinary — 0.589; Psychology, Developmental — 0.557; Education & Educational Research — 0.258; Linguistics — 0.403.

Factor 2: Social Sciences Interdisciplinary — 0.283; Education & Educational Research — 0.756; Linguistics — 0.463; Education, Scientific Disciplines — 0.515. The core has factor loadings of 0.884 for factor 1 and 0.307 for factor 2. Factor 3: Psychology, Developmental — 0.266; Philosophy — 0.778; Education, Scientific Disciplines — 0.581. If a subject area was included in several components, it was assigned to a greater factor loading. Rotation transformed in six iterations.

*Table 12* shows the most common terms corresponding to the groups of areas, differentiated by factors. Three terms were common to all areas (Vygotsky, CHAT, and learning activity), while four additional terms combined pairs of groups of areas (CHP, activity approach, university education, and sociocultural theory).

**Table 12:** Most common terms in publications of three groups of subject areas

Psychology, Multidisciplinary; Psychology, Educational; Psychology, Clinical; Social Sciences Interdisciplinary; Psychology, Developmental	Education & Educational Research; Linguistics; Psychology, Experimental	Education, ScientiThc Disciplines; Philosophy
CHP	CHAT	CHAT
Vygotsky	activity approach	Vygotsky
activity approach	Teacher education	Identity
ZPD	Vygotsky	peer assisted learning
education	CHP	University education
ASD	University education	sociocultural theory
Development	Sociocultural theory	learning activity
subjectivity	contradictions	Activity systems analysis
CHAT	learning activity	Practice-based learning
reflective-activity approach	perezhivanie	Scientific literacy
Practical knowledge	early childhood education	Context-based learning
learning activity	double stimulation	epistemology

Semantic analysis of the distributions of terms in the three groups of areas (*see Table 12*) does not reveal a clear ideological commitment to a particular school. Here, the apparent theoretical and methodological confusion between all subject areas is quite understandable since the grouping of samples of publications by subject areas or year was not appropriate for solving our task of determining the diversity of theoretical and methodological approaches. To a greater extent, however, this task correlated with the samples distributed by countries and universities. Thus, for the correct interpretation of the results, it is essential that not only that all analyses (except for distributions by years) reveal a three-cluster and three-factor structure, but also that the semantic analysis of terms (keywords) is common and distinctive for different groups of publications.

In all our terminological comparisons, we can see that some terms are included in all triads of groups or in their pairs (on average, about 20%).

**Figure 7.**
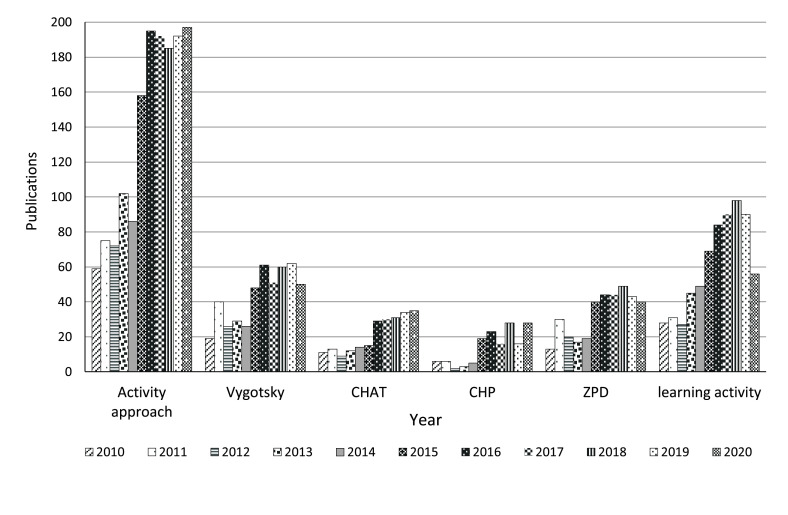
Dynamics of the use of the terms (keywords) in publications by year (according to Web of Science Core Collection)

The most frequent common terms are Vygotsky, activity approach, CHAT, CHP, ZPD, and learning activity. For this group of six terms, our additional analysis of the dynamics of their use as keywords was not limited to the framework of the sample that served as the basis of our study. *Fig. 7* depicts the dynamics of their use over an 11-year period.

If we divide this period (2010-2020) into two approximately equal sub-periods, then it is quite clear that the search terms were all used much more often in the second period than in the first one. At the same time, the undisputed leader is activity approach (the total number of publications is 1,513, peaking at 197 in 2020), followed by the learning activity (the total number of publications is 667, with the peak at 98 in 2018), and Vygotsky (the total number of publications is 472, max. 61 in 2016). The terms CHAT, CHP, and ZPD are found in less than 60 publications per year. Interestingly, the peak of the ZPD term was in 2011, while the peaks of CHAT and CHP overlap in 2016 and 2018.

## Conclusion

The present community of active cultural researchers and practitioners was formed about 40 years ago. In this context, we can observe an increase in the number of both its participants and scientific publications. This growth would have been impossible without a common cultural and historical foundation of ideas, concepts, and theories — not only psychological, but also philosophical, linguistic, semiotic, philological, pedagogical, biological, etc. (Cole, 1996; Cole, 1997; Toomela, 2016; Dafermos, 2016).

In this article, bibliometric analysis was applied to the scientific output in cultural-historical psychology. Based on journal publications from 2010 to 2020, we obtained a comprehensive overview of cultural-historical psychology-related research. Hierarchical cluster analysis and factor analysis (Principal Component Analysis) of the publications provided answers to a number of research questions, which we summarize here:

RQ1: What were the dynamics of publications in cultural-historical psychology?

The number of publications on cultural-historical psychology was established to be growing, although unevenly. The largest number of publications appeared in 2018. The minimum activity was observed from 2011 to 2014, whereas the bulk of publications came in the period from 2015 to 2019. The annual number of core publications increased 2.5 times, and the number of core+ went up more than eight times.

RQ2: Which countries, organizations and journals have contributed to cultural-historical psychology-related research?

Almost equal shares of the core+ sample were taken by publications from Russia and the United States (17.42% and 17.24%, respectively). Third place was taken by the group of authors affiliated with England (9.44%). Finland and Sweden entered the top 10 in terms of the number of publications in the core+, while Germany and Bulgaria remained below the threshhold.

Analysis of periodicals with publications of the core+ showed that more than 25% of the publications appeared in 10 journals. The leader in terms of the number of publications was the journal *Cultural-Historical Psychology* (7.26%); second place was shared by the journals *Educational Studies in Mathematics* and *Issues of Psychology* (Voprosy psikhologii) (2.9% each). Third place was taken by the *Learning Culture and Social Interaction* journal (2.72%). The top 10 journals pertained to two subject areas — Psychology (six journals) and Education & Educational Research (four journals). These journals are published by Springer (Netherlands), Taylor & Francis (England), and MSUPE (Russia) (two journals each). With regard to the geographical locations of the publishing houses of the top 10 journals, the bulk were located in England and Russia (four and three journals, respectively).

RQ3: What were the most-used keywords in the abstract section in journals on cultural-historical psychology from 2010 to 2020?

The most-used keywords reflected the prevailing areas of research such as teacher education, university education, and learning activity. Development, subjectivity, reflection, and identity were also among them, as well as ones specific to cultural-historical psychology — ZPD, perezhivanie, and double stimulation.

RQ4: What was the semantic similarity of publications from 2010 to 2020 in different countries, universities, and journals on cultural-historical psychology?

Analysis of the semantic distances of the publications in the core+ sample by the authors’ geographical affiliation showed that the following countries were the closest to each other: the United States and England; Russia and Brazil; the United States and Canada. On the other hand, the greatest distances were between Brazil and Finland; Russia and Norway; Brazil and England.

Analysis of the semantic distances between universities showed that the following universities were the closest to each other: Moscow State University of Psychology and Education and Lomonosov Moscow State University; Monash University and the University of Toronto; Moscow State University of Psychology and Education and the University of Crete. On the other hand, the largest distances were between Lomonosov Moscow State University and the University of Münich; the University of Toronto and the University of Helsinki; the University of Barcelona and the University of Helsinki.

Analysis of the semantic distances between the journals showed that the following journals were the closest to each other: *Cultural-Historical Psychology* and *Issues of Psychology*; *Cultural-Historical Psychology* and *Psychological Science and Education*; *Issues of Psychology* and *Psychological Science and Education*. On the other hand, the largest distances were between *Cultural-Historical Psychology* and *ZDM Mathematics Education*; *ZDM Mathematics Education* and *Frontiers in Psychology*; *Infancia y Aprendizaje* and *ZDM Mathematics Education*.

The most frequently used terms in all semantic groups were Vygotsky, activity approach, CHAT, CHP, ZPD, and learning activity. At the same time, the absolute leader was activity approach, followed by learning activity and Vygotsky.

In terms of further research, B.G. Meshcheryakov began work on a chronotope of cultural-historical psychology as a generalized and topological (non-metric) schematic representing the development process, similar to a genealogical tree. The units of chronotope analysis are not versions (varieties) of theory, but larger units — theoretical-methodological paradigms, and scientific schools somewhat comparable to them (Meshcheryakov, 2021). This study may provide the basis for further research.

## Supplementary Materials

To view supplementary material for this article, please visit https://doi.org/10.25449/ruspsydata.14914872.v1

## Limitations

To our knowledge, there has been no systematic analysis of the state and evolution of cultural-historical psychology using bibliometric and scientometric methods up to now. The method used in this study could help identify the historical and future development trends of research frontiers in the field of cultural-historical psychology. However, there are also some limitations in this study that can be overcome in future research.

First, we only collected data from the Web of Science Core Collection Database. Furthermore, in order to get a complete and in-depth analysis, it is preferable to significantly expand the analyzed sample of publications. Future studies can extend the search to include other databases such as Scopus.

Second, we did not differentiate the keywords according to their theoretical and methodological relevance, although it is quite obvious that keywords often include terms related to the objects of research or intervention (for example, play, education, learning, etc.). In future studies, it is possible to use different approaches to the problem of keyword selection in the bibliometric analysis, in particular, the application of the co-word method (Chen and Xiao, 2016). It should be taken into account that the results of a search query in the Web of Science Core Collection Database depend on individual user access restrictions (users may get access to data of different time periods).
